# IgM- and IgA-Enriched Intravenous Immunoglobulin Combined with Cryopreserved Human Amniotic Membrane for Pediatric Toxic Epidermal Necrolysis: A Case Report

**DOI:** 10.3390/ebj7030037

**Published:** 2026-07-08

**Authors:** Alfio Luca Costa, Alessandro Bastin, Alessandro Jad Patelli, Aurora Carnio, Vincenzo Vindigni, Franco Bassetto

**Affiliations:** Plastic, Reconstructive and Aesthetic Surgery Department, Padua University Hospital, Via Nicolò Giustiniani 2, 35128 Padua, Italy; alfioluca.costa@unipd.it (A.L.C.); alessandro.bastin@studenti.unipd.it (A.B.); alessandrojad.patelli@studenti.unipd.it (A.J.P.); vincenzo.vindigni@unipd.it (V.V.); franco.bassetto@unipd.it (F.B.)

**Keywords:** toxic epidermal necrolysis, cryopreserved human amniotic membrane, IgM- and IgA-enriched intravenous immunoglobulin

## Abstract

**Highlights:**

**What are the main findings?**

- A combined protocol achieved full, infection-free recovery in a pediatric patient with 90% TBSA toxic epidermal necrolysis.
- Staged application of cryopreserved human amniotic membrane and facial fluorescent light energy successfully induced rapid, scar-free re-epithelialization.

**What are the implications of the main findings?**

- Systemic IgM/IgA-enriched immunoglobulins offer a valuable option to prevent microbial translocation in severe skin barrier failure.
- Combining specialized tissue-bank biomaterials and non-contact biophotonics provides a reproducible workflow for pediatric critical care.

**Abstract:**

**Introduction:** Toxic epidermal necrolysis in children is a life-threatening emergency requiring prompt withdrawal of the culprit drug, transfer to an experienced center, and intensive supportive care. **Case Report:** An 11-year-old girl developed toxic epidermal necrolysis involving 90 percent of total body surface area after exposure to a nonsteroidal anti-inflammatory drug, with concomitant viral positivity. At the referring pediatric hospital, on day 2 the patient received high-dose intravenous immunoglobulin 2 g/kg and a single infusion of infliximab 5 mg/kg. She was transferred on day 6 to our Burn Unit. Histopathology confirmed complete epidermal loss. Treatment included IgM- and IgA-enriched intravenous immunoglobulin over 72 h and methylprednisolone 0.74 mg/kg/day for 30 days. Cryopreserved amniotic membrane was applied to trunk and limbs, and fluorescent light energy to the face. Complete re-epithelialization occurred within 14 days without complications. Pain resolved rapidly, with a Visual Analog Scale score of 0 on day 2. At 6 months, skin and mucosae were intact with only transient dyschromia. **Conclusions:** In this child with extensive toxic epidermal necrolysis and high predicted mortality, IgM/IgA-enriched immunoglobulin, low-dose corticosteroid and early staged cryopreserved amnion were associated with infection-free, complete re-epithelialization and full functional recovery.

## 1. Introduction

Toxic epidermal necrolysis is defined by epidermal detachment exceeding 30 percent of the total body surface area and remains one of the most severe drug-induced skin reactions across age groups. Structured causality assessment using algorithms such as Algorithm of Drug Causality in Epidermal Necrolysis is recommended whenever a culprit medication is suspected [1,2]. Severity at admission is commonly graded using Severity-of-Illness Score for Toxic Epidermal Necrolysis, a validated seven-item score that correlates with hospital mortality and helps stratify risk in specialized centers [3]. In the original series, a SCORTEN of 4 predicted a mortality of about 58 percent [3].

Intravenous immunoglobulin has been proposed as a pathophysiologically plausible treatment because pooled IgG preparations contain anti-Fas antibodies capable of blocking Fas-mediated keratinocyte apoptosis in vitro [4]. Pediatric series and reviews report rapid arrest of new blistering in many children after high-dose regimens, although effect sizes and survival advantages remain heterogeneous across cohorts [5].

IgM- and IgA-enriched preparations provide additional opsonizing and endotoxin-neutralizing properties and have been used as adjunctive therapy in sepsis and severe systemic inflammation, with dosing typically delivered over three days with monitored escalation [6,7]. These agents may be attractive in TEN, where massive epidermal loss and mucosal involvement increase the risk of translocation and shock.

For large denuded areas, cryopreserved human amniotic membrane offers a biologic cover with low immunogenicity, related to low HLA class I expression and near-absence of class II, and exerts documented anti-inflammatory, pro-migratory and pro-epithelialization effects on keratinocytes and fibroblasts [8,9,10]. Randomized and observational studies in acute burns suggest faster re-epithelialization, improved pain control and favorable scar quality compared with conventional dressings [11]. In TEN, isolated reports describe rapid epithelialization of treated areas when amnion is used as a temporary biological dressing [12].

Cryopreserved amnion is now produced under stringent quality and safety systems in specialized tissue banks, including the Treviso Tissue Bank Foundation, which has documented long-term ocular outcomes and robust traceability [13,14].

Fluorescent light energy is an emerging photobiomodulation platform that uses a chromophore gel activated by blue light to generate fluorescent emission within the visible spectrum. In a prospective series of patients with acute second-degree burns, fluorescent light energy improved healing trajectories and reduced pain without infectious complications [15].

We report a child with extensive TEN and high SCORTEN managed in our Burn Unit with a protocol combining IgM/IgA-enriched intravenous immunoglobulin, low-dose corticosteroid, early staged coverage using cryopreserved HAM and adjunct FLE on the face.

## 2. Case Report

An 11-year-old previously healthy girl (27 kg) developed fever and cough on day 0, followed within 24 h by a rapidly progressive erythematous targetoid and vesiculobullous eruption with mucosal involvement. A nonsteroidal anti-inflammatory drug had been administered shortly before the eruption. Viral testing at the referring hospital demonstrated positivity for Parvovirus B19 by PCR. No viral cultures were performed. Although concomitant viral infection may have contributed to immune activation, application of the ALDEN algorithm supported the NSAID as the most probable trigger for TEN in this patient and it was discontinued immediately [1]. Epidermal detachment progressed to approximately 90 percent of the TBSA.

At the referring pediatric hospital, on day 2 the patient received high-dose intravenous immunoglobulin 2 g/kg and a single infusion of infliximab 5 mg/kg. Despite these interventions, cutaneous denudation and mucosal erosions progressed.

**Figure 1:**
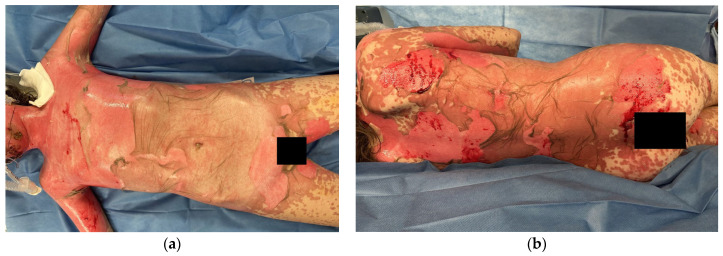
Clinical presentation on day 6 from onset of Lyell’s syndrome, the day of admission to the burn center: (**a**) anterior view; (**b**) posterior view.

On day 6 she was transferred to our Burn Unit. At admission, SCORTEN was 4, corresponding to an expected mortality of about 58 percent [3]. A first operative session was performed the same day for biopsy and gentle debridement of necrotic epidermis. Superficial dermal areas were dressed with medical-grade honey gauze. After surgery, she was admitted to the pediatric intensive care unit and managed with continuous intravenous sedation for analgesia (Figure 1).

Histopathologic examination of a skin biopsy demonstrated complete loss of the epidermis with full-thickness epidermal necrosis and detachment at the dermoepidermal junction. The underlying dermis showed lymphangiectasia and sparse chronic interstitial and perivascular inflammatory infiltrates. No vasculitis, significant eosinophilic infiltrate, or histologic features suggestive of an alternative blistering disorder were identified. These findings were consistent with toxic epidermal necrolysis.

At admission, ophthalmologic evaluation demonstrated mild ocular involvement, including eyelid epithelial loss, conjunctival hyperemia, rare pseudomembranes, and a transient central corneal epithelial defect. Treatment was initiated with topical amniotic membrane eye drops five times daily and ofloxacin ophthalmic ointment three times daily in both eyes, followed by topical antibiotic–steroid therapy (tobramycin/dexamethasone eye drops).

Given ongoing progression despite high-dose IVIg and infliximab and the absence of evidence supporting additional TNF-α blockade in this setting, infliximab was discontinued at admission. We initiated an IgM- and IgA-enriched human immunoglobulin (Pentaglobin, Biotest, Dreieich, Germany) regimen according to product characteristics, infused over 72 h at 5 mL/kg/day with stepwise ramp as tolerated. No infusion reactions occurred. Methylprednisolone 0.74 mg/kg/day was started the same day and maintained for 30 days, then tapered. No systemic antibiotics were administered apart from standard perioperative cefazolin prophylaxis for operative sessions. From admission until coverage with amnion, wound care relied on medical-grade honey gauze.

**Figure 2:**
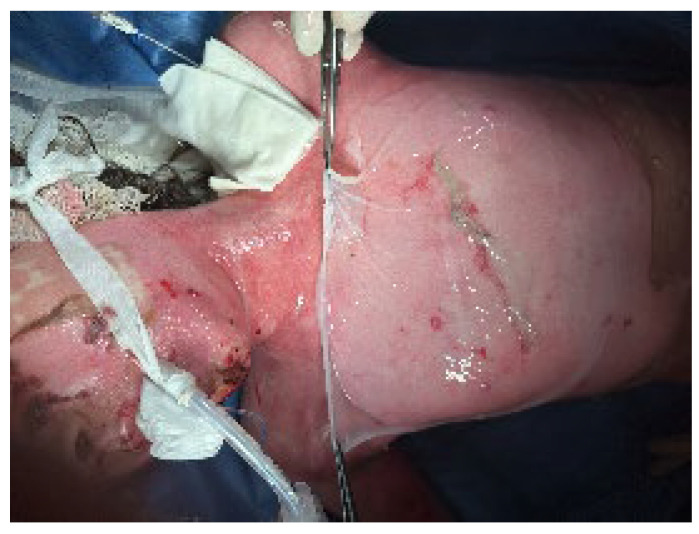
Day 9 from onset. Application of cryopreserved human amniotic membrane.

On day 9 an operative session under general anesthesia was performed for biological coverage. Cryopreserved HAM was procured from the Treviso Tissue Bank Foundation, a certified non-profit regional bank operating under European and national standards for procurement, processing and distribution of human tissues [13,14]. Multiple amniotic sheets were applied epithelial side up to the anterior trunk and both upper limbs. The sheets were secured with metallic clips placed at the periphery. Petrolatum-impregnated gauze was used as the only secondary dressing on amnion-covered areas (Figure 2).

On day 11 a second operative session completed HAM coverage of the posterior trunk, gluteal and inguinal regions using the same technique. During this procedure, esophagogastroduodenoscopy was performed because of persistent odynophagia. Endoscopy revealed edematous, spontaneously bleeding oral mucosa; an erythematous proximal esophagus with fibrin deposition and mild edema; and at the cardia, partial mucosal discontinuity with thin pseudomembranes. No active bleeding or perforation was observed.

The facial skin showed only patchy superficial denudation. For these reasons, we selected FLE to treat the face. Treatment consisted of application of the chromophore gel followed by illumination with a multi-LED lamp at the manufacturer’s recommended distance and duration, in association with gentle topical care, mirroring protocols reported for acute burns [15].

**Figure 3:**
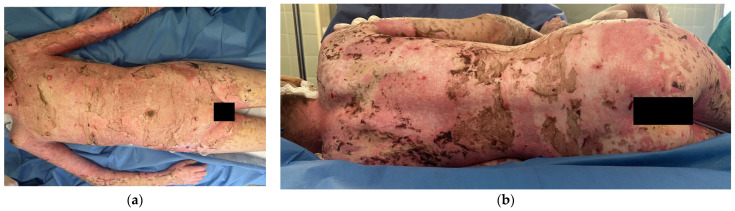
Day 21 from onset (day 10 after initial HAM placement). Integration of amniotic membranes on trunk and upper limbs with islands of neoepithelium: (**a**) anterior view; (**b**) posterior view.

After complete HAM coverage, petrolatum-impregnated gauze remained the only secondary dressing on amnion-treated areas. Scheduled operative dressing sessions took place on days 16, 19 and 23. At each session we inspected HAM integration, trimmed loose margins and renewed petrolatum gauze. Metallic clips were removed in the operating theater 14 days after the last amnion application (Figure 3).

Re-epithelialization progressed steadily across trunk and upper limbs. Full re-epithelialization of HAM-covered areas occurred about 14 days after the first amnion placement. Throughout the course there were no septic episodes. Pain scores were not scorable during the phase of deep sedation; after reduction in sedation following HAM coverage, the patient reported Visual Analog Scale 0 on post-HAM day 2.

**Figure 4:**
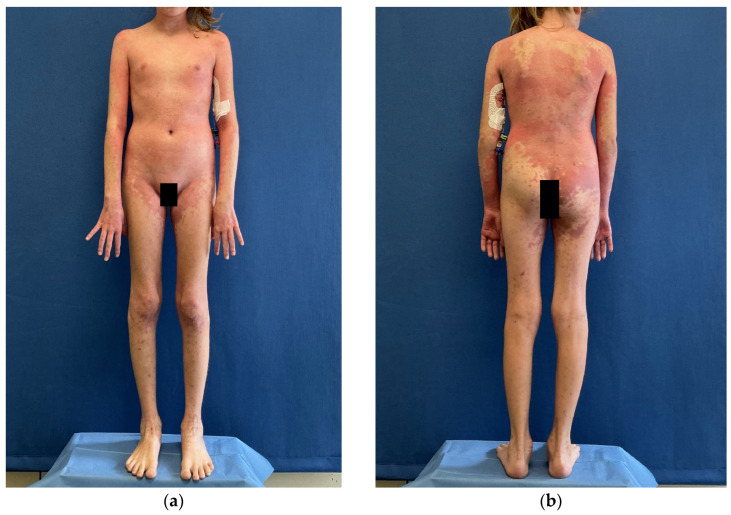
Day 27 from onset. Complete re-epithelialization, 18 days after the first human amniotic membrane application, corresponding to the day of discharge in good general condition: (**a**) anterior view; (**b**) posterior view.

She was discharged home from the Burn Unit in good condition after 20 days from the first HAM coverage (Figure 4).

At the 6-month follow-up, skin and mucosae were intact, with only transient dyschromia and no functional sequelae on range of motion and daily activities; ophthalmologic follow-up confirmed complete resolution of ocular involvement without corneal, conjunctival, or eyelid sequelae.

## 3. Discussion

This case describes a practical pathway for extensive pediatric TEN integrating drug withdrawal, early transfer to a specialized Burn Unit, systemic immunomodulation with an IgM/IgA-enriched preparation and low-dose corticosteroid, and early biologically active coverage with cryopreserved HAM, with adjunct FLE on the face. Several aspects merit comment.

First, the initial management at the referring center was aligned with current concepts. ALDEN scoring supported a probable causality link to the NSAID, which was stopped promptly [1]. High-dose IVIg 2 g/kg on day 2 is biologically plausible in TEN, given in vitro evidence that pooled immunoglobulin preparations inhibit Fas-mediated keratinocyte apoptosis [4], and pediatric series reporting rapid arrest of new lesions in many children [5]. The lack of early clinical response in this patient illustrates the variability of outcomes observed in the IVIg literature.

Upon admission to our Burn Unit, SCORTEN was 4, placing the child in a group with a predicted mortality of approximately 58 percent [3]. The high SCORTEN underlines the severity of this case.

Second, we added an IgM- and IgA-enriched intravenous immunoglobulin preparation and low-dose corticosteroid as part of our internal protocol. IgM/IgA-enriched immunoglobulins have been associated with improved hemodynamics, microcirculation and pathogen clearance in septic and hyperinflammatory states when administered over three days with dose escalation and close monitoring [6,7]. In a setting such as extensive TEN, where skin barrier failure and mucosal denudation create a high-risk interface for microbial translocation, these additional opsonizing and endotoxin-neutralizing properties are attractive. In our patient the regimen was well tolerated and no infusion-related adverse events occurred. The choice of a modest daily dose of methylprednisolone reflected a balance between potential benefits on inflammation and the risk of impaired wound healing or sepsis.

Third, the early staged application of cryopreserved HMA addressed three priorities for denuded pediatric skin: protection and moisture balance, analgesia and promotion of epithelial gap closure. Amnion has very low expression of HLA class I and minimal class II and contains an extracellular matrix and growth factor milieu that supports keratinocyte migration and proliferation, modulates TGF-β signaling and attenuates myofibroblast differentiation [8,9,10]. Experimental work has documented activation of ERK and JNK pathways, enhanced re-epithelialization and a more favorable remodeling profile [9]. Clinical studies in acute burns report faster re-epithelialization and reduced pain compared with conventional dressings [11]. Case reports in TEN suggest accelerated closure of treated areas when HAM is used as a biologic dressing [12]. Our experience supports these signals. After completion of HAM coverage on days 9 and 11, the patient showed rapid progression of islands of neoepithelium, no septic complications and complete re-epithelialization within approximately 14 days. The analgesic effect was clinically evident, with VAS 0 shortly after coverage. The absence of infection over prolonged denudation is noteworthy, although it cannot be attributed solely to amnion and likely reflects the combination of barrier function, meticulous supportive care and systemic immunomodulation.

Fourth, FLE was chosen for the face rather than HAM. FLE has been shown in a prospective case series and ex vivo work to accelerate healing of acute second-degree burns, reduce pain and modulate inflammatory and remodeling pathways, including regulation of MHC class II molecules and induction of pro-regenerative mediators such as FGF2 and TGF-β isoforms [15]. In our patient, facial epithelialization progressed uneventfully with FLE and gentle topical care, without the need for grafting or secondary reconstructive procedures. While our single experience cannot validate FLE in TEN, it suggests that this non-contact modality can be integrated safely into a complex multimodal regimen.

Upper gastrointestinal endoscopy performed during the second HAM session documented mucosal involvement of the oral cavity, proximal esophagus and cardia. In addition, ophthalmologic evaluation revealed mild ocular surface involvement characterized by conjunctival hyperemia, eyelid epithelial loss, rare pseudomembranes and a transient corneal epithelial defect. This confirms the systemic nature of TEN and underscores the need for multidisciplinary collaboration.

Several limitations must be acknowledged. This is a single case, so no causal inference can be made regarding the relative contribution of IgM/IgA-enriched immunoglobulin, corticosteroid, HAM or FLE. Treatment components were instituted in close temporal sequence, and supportive care in the Burn Unit and PICU likely played a major role in the favorable outcome. The prior administration of high-dose IVIg and infliximab at the referring center may also have influenced disease biology. Moreover, we did not perform mechanistic investigations aimed at characterizing the immunopathology of TEN in this patient. Such studies could have included serum biomarker analyses (e.g., cytokines, granulysin, or soluble Fas ligand), immunophenotyping of circulating immune cells, or immunohistochemical and molecular characterization of inflammatory pathways in skin biopsy specimens. These investigations were not part of routine clinical management at our institution and were therefore not obtained during the acute phase of illness. Consequently, our discussion of the potential biological effects of IgM/IgA-enriched immunoglobulin, corticosteroids, HAM, and FLE relies on previously published experimental and clinical studies rather than on patient-specific mechanistic data [4,5,6,7,8,9,10,11,15]. Finally, the prognostic significance of the SCORTEN value reported in this case should be interpreted with caution, as recent studies suggest that the score may overestimate mortality in contemporary specialized settings and its performance in pediatric populations remains less well defined [16,17].

Despite these caveats, the coherence between the high baseline SCORTEN, the absence of infection, the relatively rapid and complete re-epithelialization and the literature on IgM/IgA immunoglobulins and HAM supports the plausibility of this integrated approach. Additional pediatric case series and multicenter registries will be needed to refine protocols and clarify where such interventions add most value.

## 4. Conclusions

In an 11-year-old girl with extensive TEN, high SCORTEN and progression despite early high-dose IVIg and infliximab at a referring center, our Burn Unit protocol combining IgM/IgA-enriched immunoglobulin, low-dose corticosteroid, staged cryopreserved HAM coverage and FLE for patchy facial lesions resulted in infection-free, complete re-epithelialization and full functional recovery at 6 months.

Centers with access to pediatric intensive care and certified tissue banking may consider a similar pathway, carefully documenting SCORTEN at admission, timelines of immunomodulation and biologic coverage, and pain and functional outcomes in order to inform future comparative studies.

## Data Availability

The original contributions presented in this study are included in this article. Further inquiries can be directed to the corresponding author.

## Abbreviations

The following abbreviations are used in this manuscript:

TEN	Toxic Epidermal Necrolysis
ALDEN	Algorithm of Drug Causality in Epidermal Necrolysis
SCORTEN	Severity-of-Illness Score for Toxic Epidermal Necrolysis
HAM	Human Amniotic Membrane
FLE	Fluorescent light energy
NSAID	Nonsteroidal Anti-Inflammatory Drug
IVIg	Intravenous Immonoglobulin
VAS	Visual Analog Scala
